# Effects of Natural Brown Cotton Bleached Gauze on Wound Healing

**DOI:** 10.3390/ma15062070

**Published:** 2022-03-11

**Authors:** Jingying Xu, Miao Su, Zimin Jin, Wenlong Zhou, Yuqiang Sun, Youyi Jin, Zhansong Shi

**Affiliations:** 1 College of Textile Science and Engineering, Zhejiang Sci-Tech University, Hangzhou 310018, China; a785436476@163.com (J.X.); kivenjin@163.com (Z.J.); wzhou@zstu.edu.cn (W.Z.); 2 College of Life Sciences and Medicine, Zhejiang Sci-Tech University, Hangzhou 310018, China; sunyuqiang@zstu.edu.cn; 3 Zhejiang Jiu Shun Textile Co., Ltd., Lanxi 321104, China; zjcljyy@126.com; 4 Zhejiang Yunshan Textile Printing Dying Co., Ltd., Lanxi 321100, China; shi8863007@126.com.cn

**Keywords:** natural colored cotton, medical gauze, wound healing, medical material

## Abstract

Natural brown cotton has favorable antibacterial and antioxidant properties. In this study, we explored the effect of gauze made from natural brown cotton after scouring and bleaching on wound healing in rats. In this work, a control experiment was adopted. The control group used absorbent cotton gauze, and the experimental group utilized natural brown cotton bleached gauze. The materials were applied to rat models to explore the effects of the two dressings on wound healing. By analyzing the wound healing state of rats, calculating the healing rate, and combining the pathological HE staining, Masson staining, and CD31 immunohistochemical staining, the results showed that both gauzes have positive effects on the wound healing of the rats. Moreover, compared with the control group, the wound healing rate of rats in the experimental group increased by 14.81%, the number of inflammatory cells decreased by 12.93%, the number of new blood vessels increased by 6.88%, the growth rate of the granulation tissue area was 10.76%, the step-up rate of the area occupied by collagen was 33.71%, and the increase rate of optical density value was 10.00%. This study found that natural brown cotton bleached gauze has a better effect on wound healing than ordinary absorbent cotton gauze, and can be used as medical dressings.

## 1. Introduction

Natural colored cotton first cultivated in Peru 4000 years ago has existed since ancient times [1]. Through the use of cytogenetics and archeology, it can be traced back to the cultural center of Central America and the Andes on the west coast of South America 5000 years ago [2]. A kind of natural colored cotton was planted in ancient China. It was woven into a “purple flower cloth” by farmers in the south of the Yangtze River. However, due to the small output of colored cotton, short fibers, and the invention and development of chemical dyes, colored cotton products disappeared [3]. The Soviet Union first began to study colored cotton in the early 1950s. The main colors included brown, green, red, cyan, blue, and black [4]. At present, the research on natural colored cotton is concentrated in the field of agricultural biotechnology and textile and clothing. The research directions include new natural colored cotton line cultivation and industrial planting, pigment separation and component identification, and colored cotton fabric and clothing product development [5,6].

In the field of agricultural biotechnology, Chen adopted a new cross-matching model to change the traditional breeding ideas, using dark brown colored cotton germplasm as the female parent and white cotton germplasm as the male parent, and successfully bred new high-quality colored hybrid cotton varieties [7]. Many researchers have devoted their work to the identification of natural colored cotton pigments. Due to the different methods of pigment extraction, separation, and purification, the obtained components are also not the same. Ren summarized the possibilities of natural brown cotton pigments into four types: catechin, flavonoids, quinones, and proanthocyanidins [8]. Ryser believes that the brown color of brown cotton linters is caused by tannin precursors catechins and tannin derivatives. At the same time, it is speculated that the color of brown fibers may be related to the tannins derived from catechins in the vacuoles [9,10]. Zhao and Wang extracted brown cotton fiber pigments, which may be flavonoids, with methanol at room temperature [11]. By comparing the maturation process of white cotton and colored cotton fiber, Hua found that colored cotton consumes many carbohydrates in this process, which may be used to synthesize flavonoids [12]. Zhan used ultraviolet-visible and infrared spectroscopy and assumed that the unique compound formed by oxidation of tannins are responsible for the pigments in brown cotton [13]. Through the vanillin–hydrochloric acid method and Borntrager’s test, Feng believes that the biosynthesis of proanthocyanidins is the key process of brown cotton fiber pigmentation, and quinones may be the main pigments that form the color brown [14]. Li found that proanthocyanidin is the main component of the pigment substance of brown cotton fiber by comparing the proteomic analysis of brown cotton fiber and its near equivalent to white cotton fiber [15]. Zhen believes that the pigment of natural brown cotton fiber is caused by condensed tannins formed by the oxidation of proanthocyanidins, but the specific role of proanthocyanidin biosynthesis and sugar metabolism in brown pigmentation during fiber development is unclear [16].

Fan found that proanthocyanidin B2 (PCB2) improves endothelial progenitor cell function and promotes wound healing in diabetic mice by activating Nrf2. PCB2 treatment accelerates wound healing in diabetic mice and increases angiogenesis, perhaps by improving EPC mobilization and function to mediate [17]. Badeggi synthesized gold nanoparticles (Au NPs) with the total extract of broad bean and proanthocyanidins and fully characterized them. They not only revealed the ability of proanthocyanidin dimers to form biological stability and biologically active Au NPs, but also significantly to enhance the activity of natural products, which can improve intelligent delivery in future biomedical applications [18]. Wang reported on natural antioxidant proanthocyanidins extracted from sea buckthorn and laboratory-made apocynum cellulose nanofibers as core drugs. The slow-release nanofiber membrane was prepared by electrostatic spinning on polylactic acid/polyvinylpyrrolidone nanofibers. The results showed that the extracted proanthocyanidins are successfully encapsulated in the core–sheath structure nanofibers and had high antioxidant activity [19].

With the deepening of research, the excellent properties of natural brown cotton have been discovered, especially its antibacterial and antioxidant properties [20,21]. These two characteristics show that natural colored cotton has great potential in the application of medical and sanitary materials, making it a possible material to be used in medical gauze.

Based on the related research on the pigment composition of natural brown cotton and the biological application of proanthocyanidins, we used a controlled experiment to explore whether bleached brown cotton fabric can be used as a wound dressing. Natural brown cotton can be obtained through natural planting and has antibacterial and antioxidant properties. After desizing and bleaching, it can promote the healing of wounds, which makes it possible to be applied to medical materials. This research prompts new ideas for the field of medical gauze materials and provides a reference basis for follow-up research.

## 2. Materials and Methods

### 2.1. Gauze Material

**Figure 1:**
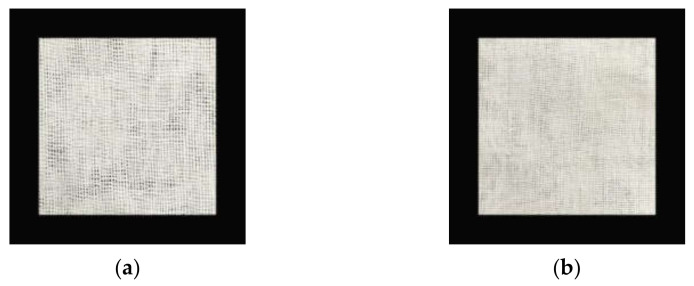
Two dressings. (**a**) Conventional cotton gauze used for control group; (**b**) brown cotton bleached gauze used for the experimental group.

The material used in this study was fabric made of natural brown cotton. The fabric was subjected to sodium hydroxide scouring and hydrogen peroxide bleaching to obtain natural brown cotton for experiment bleached gauze. The control group was set as absorbent cotton gauze, and the experimental group was set as natural brown cotton bleached gauze. Before the animal experiment, the gauze of the control group and the experimental group were sterilized by pulsating vacuum steam. The gauze samples are shown in Figure 1.

### 2.2. Experimental Protocol for Wound Healing in SD Rats

#### 2.2.1. Laboratory Animals and Housed Conditions

Eighteen male SD rats, weighing 180–220 g, SPF grade, were purchased from Shanghai Slack Laboratory Animal Co., Ltd. SCXK (Shanghai, China) 2017-0005, certificate number: 20170005049788. The license number of the laboratory animal room was SYXK (Zhejiang, China) 2020-0013. The drinking water for feeding rats was sterilized second-level ultrapure water, and the quality met the requirements of GB5749-2006 “Sanitary Standards for Drinking Water”. The rats were reared in a temperature range of 20–25 °C and a relative humidity range of 40–70%. They were acclimatized in the animal room environment for one week before the test. The maintenance feed was provided by Jiangsu Synergy Pharmaceutical and Biological Engineering Co., Ltd. (Nanjing, China), and the standard GB14924.3-2010 “Nutrient Composition of Compound Feed for Laboratory Animals” was implemented.

#### 2.2.2. Reagents and Equipment

The reagents and equipment used in the experiment are shown in Table 1 and Table 2.

#### 2.2.3. Experimental Method

**Figure 2:**
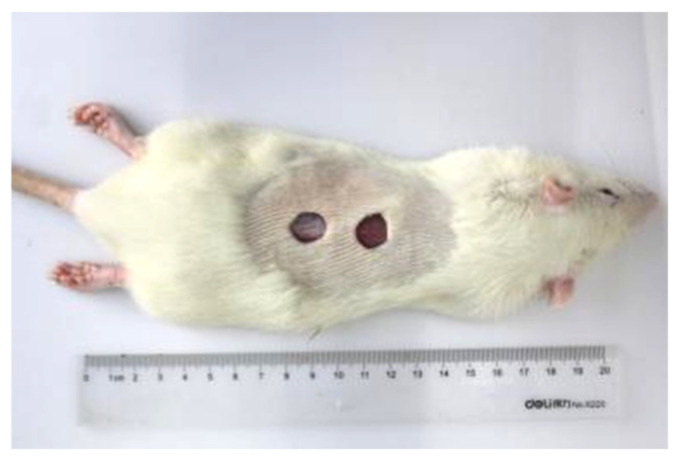
Construction of rat wound model.

**Figure 3:**
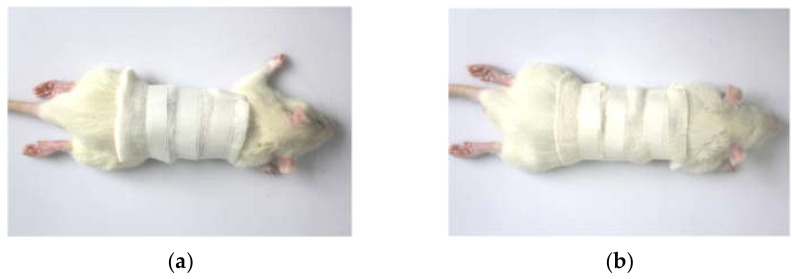
We wrapped the rats’ wound with two types of gauze: (**a**) conventional cotton gauze used for rats in the control group, and (**b**) brown cotton bleached gauze used for the experimental group.

Establishment of rat wound model: Eighteen rats were divided into two groups and fed adaptively in the experimental animal room for one week. For anesthesia, 30–50 mg/kg sodium pentobarbital 1% was injected intraperitoneally. The hair on the back of the rat was shaved and the superficial skin was disinfected. A circular full-thickness wound with a diameter of 10 mm was taken on the back of the rat up to the fascia, and the skin was removed by stainless steel puncture [22]. There were two wounds on each rat, as shown in Figure 2. Then, the rats were reared in different cages after being wrapped with two dressings, as shown in Figure 3. Pictures of the wounds were taken at each dressing change. The wound healing rate was calculated using ImageJ 1.46 software image analysis. After opening the software, we imported the images of wounds, selected the composition shape consistent with the wound, and calculated the average value. Statistical analysis was performed with SPSS 20.0 statistics, and one-way analysis of variance was used. The comparison between groups was performed by *t*-tests, and *p* < 0.05 was considered statistically significant. The formula for calculating the wound healing rate is as follows:

$$\mathrm{Wound}\;\mathrm{healing}\;\mathrm{rate}\;(\%)=\frac{\mathrm{Initial}\;\mathrm{area}-\mathrm{Observation}\;\mathrm{point}\;\mathrm{area}}{\mathrm{Initial}\;\mathrm{area}}\times 100\%$$

Injured tissue collection: The rats’ dressings were changed every 2 days, and the wound status of the rats was recorded by taking pictures. On the 3rd, 7th, and 14th days, three rats in each group were sacrificed, and the wound tissues were collected. The repair tissue (about 1.5 cm in diameter) from the edge of the wound to the center of the wound was taken, including part of the normal skin. Four percent polynail liquid fixative was used for fixation.

Paraffin section preparation: The rat wound tissue involved in this experiment was sectioned with paraffin section. The tissue was embedded with paraffin blocks for subsequent pathological examination. On the 3rd, 7th, and 14th days, 12 paraffin blocks (6 in the control group and 6 in the experimental group) with damaged tissue were mounted for HE staining, Masson staining, and CD31 staining.

HE staining: In order to further observe the repair of internal cells and tissues of the injured skin of SD rats, sections of the wound were stained. Dewaxing was performed by successively immersing the samples in xylene (I) for 15 min, xylene (II) for 15 min, 100% alcohol (I) for 1 min, 100% alcohol (II) for 1 min, 95% alcohol for 1 min, 80% alcohol (I) for 1 min, and tap water for 1 min. Then, the stain sections were immersed in hematoxylin dye solution for 5 min at room temperature and washed with tap water for 1 min, immersed in 1% hydrochloric acid alcohol solution for a few seconds and then tap water until the tissue turned blue, and dyed with eosin dye solution for 5 min. Finally, the floating color of the slide was washed away by using tap water. Then, the samples were dehydrated, transparent, and sealed by immersing them in 80% alcohol for 30 s, 95% alcohol (I) for 30 s, 95% alcohol (II) for 30 s, 100% alcohol (I) for 30 s, 100% alcohol (II) for 30 s, and xylene (I) transparent for 3 min, and sealed with natural gum.

Masson staining: Tissues were fixed with 10% formalin and routinely dehydrated and embedded. Sections were 4 μm thick and dewaxed in water. The sections were placed in Bouin’s solution, placed in a 60 °C incubator for 2 h for mordant staining, and then rinsed with running water until the yellow color on the sections disappeared. The lapis lazuli staining solution was added dropwise for 3 min and then washed with water. Mayer’s hematoxylin staining solution was added dropwise for 3 min, followed by washing with water. The acidic ethanol differentiation solution was differentiated for several seconds and rinsed with running water for 10 min, followed by staining dropwise with ponceau acid fuchsin staining solution for 10 min, then rinsing with distilled water. After the phosphomolybdic acid solution was treated for about 10 min, the supernatant was poured into the aniline blue staining solution for 5 min. After treatment with weak acid solution for 2 min, 95% ethanol was rapidly dehydrated. Dehydration with absolute ethanol was carried out 3 times for 10 s each time. Xylene was transparent 3 times, 2 min each time. Finally, they were sealed with neutral glue. The staining morphology of tissue sections was observed through a microscope, and the amount of collagen growth was calculated according to the formula:

$$\mathrm{Area}\;\mathrm{ratio}\;\mathrm{occupied}\;\mathrm{by}\;\mathrm{collagen}\;\mathrm{fibers}=\frac{\mathrm{Collagen}\;\mathrm{fiber}\;\mathrm{area}}{\mathrm{The}\;\mathrm{total}\;\mathrm{area}}\times 100\%$$

CD31 immunohistochemical staining: Tissues were dewaxed with xylene (I) for 20 min, xylene (II) for 20 min, then rehydrated with 100% ethanol (I) for 5 min, 100% ethanol (II), 95% ethanol for 5 min, and 80% ethanol for 5 min. Tissues were then washed 3 times with PBS for 3 min each. To block and inactivate endogenous peroxidase, 3% H_2_O_2_ was used to incubate the samples at 37 °C for 15 min, and then they were rinsed with PBS 3 times for 3 min each. The samples were prepared in 0.01 M citrate buffer (pH = 6.0) and boiled in water, and antigen retrieval was carried out for 10 min. The samples were then cooled to room temperature naturally, and washed 3 times with PBS for 3 min each time. We added the primary antibody and incubated the samples in the refrigerator at 4 °C overnight, transferred them to room temperature for 30 min, and rinsed them with PBS 3 times, for 5 min each time. Then, we dropped in the secondary antibody, incubated the samples at 37 °C for 30 min, and rinsed them with PBS 3 times, for 5 min each time. DAB reaction staining was performed, the reaction progress was observed under a microscope, and the samples were fully rinsed with tap water.

## 3. Results and Discussion

### 3.1. Analysis of Wound Healing in SD Rats

**Figure 4:**
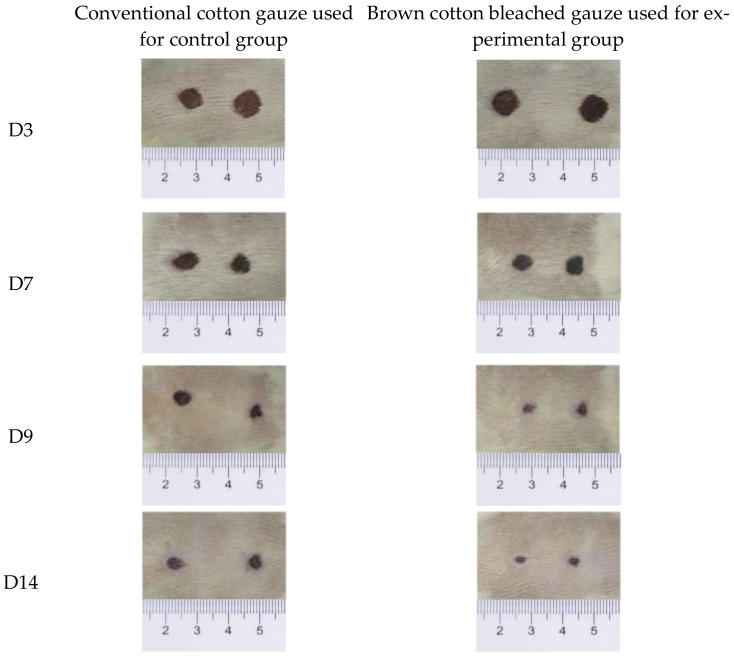
Wound healing status on D3, D7, and D14.

Figure 4 shows the wound healing on the 3rd, 7th, and 14th days.

**Figure 5:**
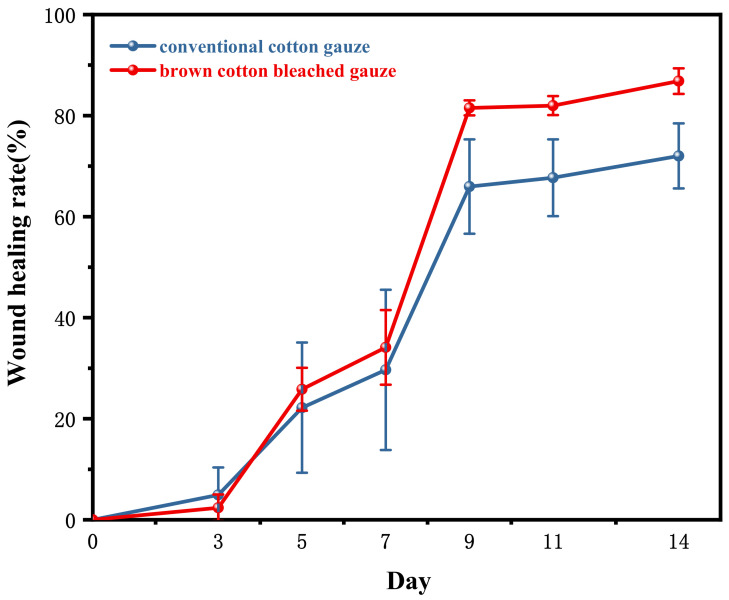
Wound healing rate in rats.

At the end of the experiment, the healing rate (%) was statistically significant in the results of one-way analysis of variance. The healing rate of the control group was 72.02 ± 6.44%, while that of the experimental group was 86.83 ± 2.53% (*p* < 0.01). The experimental results are shown in Table 3 and Figure 5.

### 3.2. Wound Histological Analysis

**Figure 6:**
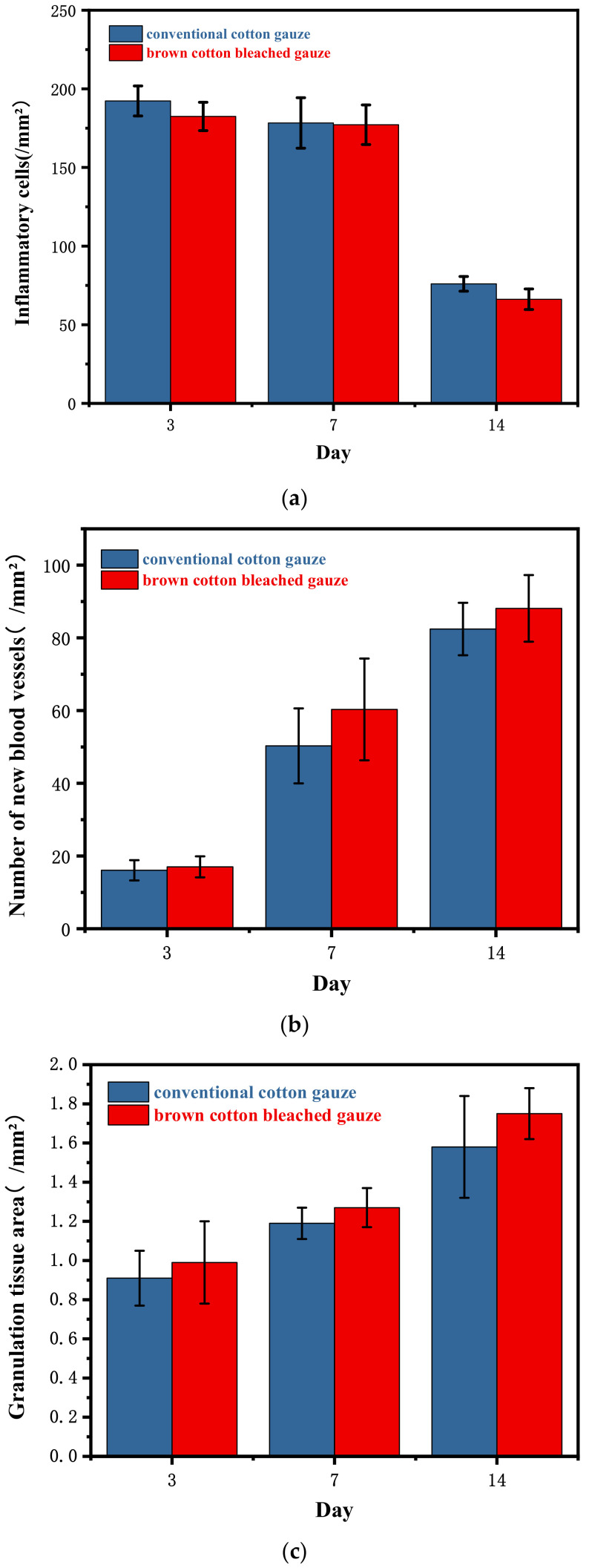
The number of inflammatory cells (**a**) and new blood vessels (**b**), and the area of granulation tissue (**c**).

**Figure 7:**
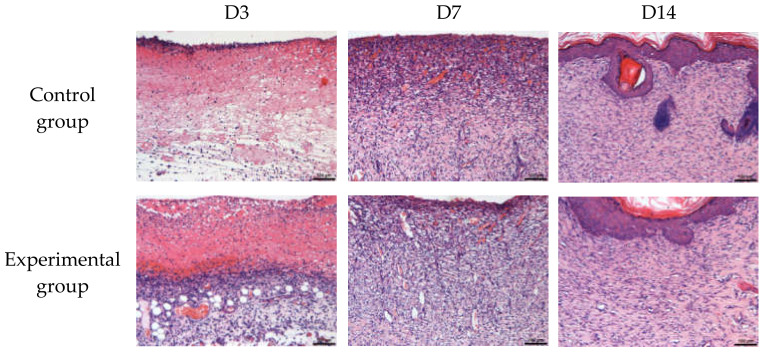
HE staining results.

HE staining analysis: Inflammatory cells, new blood vessels, and granulation tissue were observed 10 times. In the present study, better wound repairing effects were observed in the natural brown cotton bleached gauze experimental group than the control group. In the same group, we observed notable inflammation and congestion at 3 days, and fewer new blood vessels. At 7 days, inflammatory cells decreased, new blood vessels increased, and granulation tissue was formed. The wound was basically repaired in 14 days, inflammatory cells decreased in a large area, and new blood vessels increased. The purple stained cells are inflammatory cells, and the long cells are fibroblasts (used to secrete collagen). Table 4 and Figure 6 show the number of inflammatory cells, the number of new blood vessels, and the area of granulation tissue in each group. As can be seen from Figure 7, the purple endothelial cells on the periphery wrap the red new blood vessels inside, and the two are wrapped in clusters to form granulation tissue. Figure 7 shows the results of HE staining.

Masson staining analysis: The area occupied by collagen was observed at a 10-fold scale. Red staining is collagen type 1 and blue is collagen type 3 (scar tissue), observed under the microscope for comprehensive pathological description and raw data analysis.

**Figure 8:**
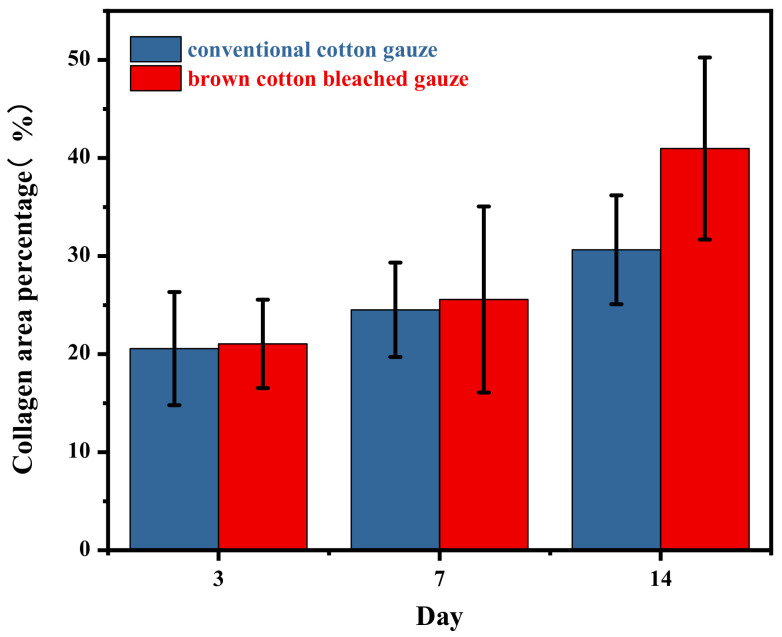
The area ratio occupied by collagen.

**Figure 9:**
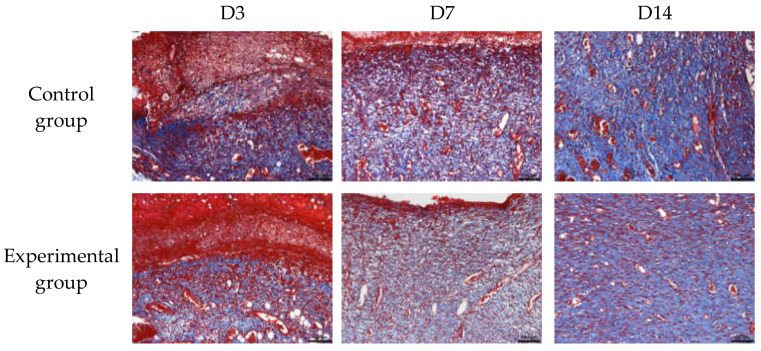
Masson staining results.

The collagen in the control group occupied fewer areas than the natural brown cotton bleached gauze for the experimental group at different times during the experiment. In the same group, the area occupied by collagen on day 14 was significantly different from that on day 3 and day 7. Table 5 and Figure 8 show the area ratio occupied by collagen, and Figure 9 shows the results of Masson staining.

**Figure 10:**
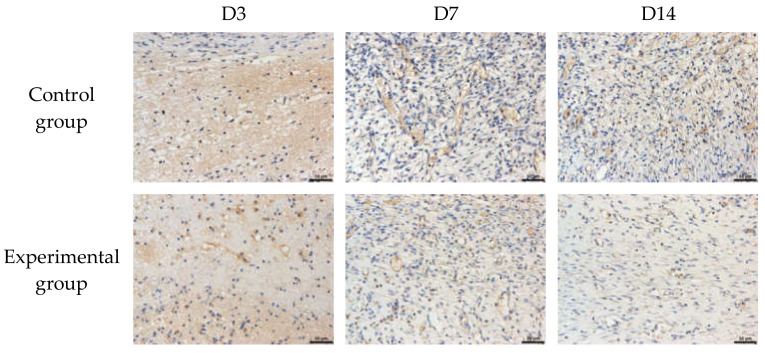
CD31 immunohistochemical staining results.

CD31 immunohistochemical analysis: HE staining is a general view of the overall situation. Immunohistochemistry looks at the formation of new blood vessels in detail. Histochemical staining reflects the presence of proteins secreted by platelet endothelial cells. Identified through the target protein, the brown staining indicates the new blood vessels. The expression of CD31 in skin tissue was observed at a magnitude of 20×. At the same time, the positive expression in the conventional cotton gauze was weaker than the natural brown cotton bleached gauze. In the same group, the positive expression was the strongest at 14 days, followed by 7 days, and the weakest at 3 days. Table 6 represents the optical density value and Figure 10 shows the results of CD31 immunohistochemical staining.

The results of the study show that the two kinds of gauze can heal wounds in rats. On the 14th day, the healing rates of the control group and the experimental group were 72.02% and 86.83%, respectively (*p* < 0.05). Inflammatory cells totaled 76.01 ± 4.68/mm^2^ and 66.18 ± 6.54/mm^2^ (*p* < 0.01) and new blood vessels amounted to 82.44 ± 7.22/mm^2^ and 88.11 ± 9.17/mm^2^ (*p* > 0.05). The areas of granulation tissue were 1.58 ± 0.26/mm^2^ and 1.75 ± 0.13/mm^2^ (*p* > 0.05). The area ratios of collagen were 30.64 ± 5.55% and 40.97 ± 9.29% (*p* < 0.05). The optical density values were 0.20 ± 0.02 and 0.22 ± 0.03 (*p* > 0.05).

We believe these results may be due to the antibacterial and antioxidant properties of the brown cotton bleached gauze. Combining the number of inflammatory cells in HE staining and the collagen volume fraction in Masson staining, we determined that the inflammatory cells in the control group and the experimental group amounted to 76.01 ± 4.68/mm^2^ and 66.18 ± 6.54/mm^2^, respectively (*p* < 0.01). Compared with the experimental group, the proportion of collagen was 30.64 ± 5.55 and 40.97 ± 9.29 (*p* < 0.05), indicating that the brown colored cotton bleached gauze had a significant effect on reducing wound inflammation and promoting collagen deposition. This may be due to the antibacterial properties of the brown cotton bleached gauze, which reduces the chance of bacterial infection and prevents other harmful substances from entering the wound. Moreover, its antioxidant properties can remove excess reactive oxygen species generated in the wound, and increase the degree of cross-linking with collagen to promote collagen production. At the same time, brown cotton bleached gauze can also help to form new blood vessels and granulation tissue to some extent. The brown cotton bleached gauze in the experimental group can promote the formation of new blood vessels, granulation tissue, and collagen in the wound; reduce the number of inflammatory cells; and improve the wound healing rate.

## 4. Conclusions

The observations from this study suggest that brown cotton bleached gauze is effective in an animal model of wound healing. It can significantly improve the wound healing rate, reduce the number of inflammatory cells, and increase collagen deposition. It also aids in the formation of new blood vessels and granulation tissue. Brown cotton bleached gauze, which can accelerate wound healing and improve cost-effectiveness, has potential as a wound care material for future development, and can hopefully be produced in the market like absorbent cotton gauze. This paper is the first exploratory study in this direction, and follow-up research will start with the establishment of animal models to explain the mechanism of natural brown cotton bleached gauze to accelerate wound healing.

## Figures and Tables

**Table 1:** Experimental reagents.

Experimental Reagent	Manufacturer
Hematoxylin	SIGMA
Eosin	SIGMA
Improved Masson Tricolor Staining Solution	SOLARBIO
CD31 Antibody	GeneTex
(HAMA) ELISA Kit	Beijing Zhongshan Golden Bridge Biotechnology Co., Ltd.

**Table 2:** Laboratory equipment.

Experimental Equipment	Model	Manufacturer
Pulsating vacuum pressure steam sterilizer	MZQD-0.6C	Wuhan Jianghan Medical Pharmaceutical Equipment Co., Ltd.
Rotary microtome	RM2235	LEICA
Histopathology bake instrument drift	TEC2500	Changzhou Hao Silin Instrument Equipment Co., Ltd.
Microscope	BX43	OLYMPUS
Waterproof constant temperature incubator	PYX-DHS500BS-Ⅱ	Shanghai Yuejin Medical Equipment Co., Ltd.

**Table 3:** Wound healing rate in rats ($\bar{x}$ s ± d).

Group	D0	D3	D5	D7	D9	D11	D14
Control group	0.00 ± 0.00	4.91 ± 5.46	22.19 ± 12.89	29.68 ± 15.86	65.96 ± 9.34	67.70 ± 7.59	72.02 ± 6.44
Experimental group	0.00 ± 0.00	2.38 ± 2.64	25.83 ± 4.25	34.12 ± 7.38	81.53 ± 1.48	81.98 ± 1.88	86.83 ± 2.53 **

Note: Compared with the control group, ** *p* < 0.05.

**Table 4:** The number of inflammatory cells and new blood vessels, and the area of granulation tissue.

	Inflammatory Cells (/mm^2^)	New Blood Vessels (/mm^2^)	Area of Granulation Tissue (/mm^2^)
Control group-3d	192.29 ± 9.56	16.07 ± 2.79	0.91 ± 0.14
Experimental group-3d	182.46 ± 9.03	17.02 ± 2.89	0.99 ± 0.21
Control group-7d	178.30 ± 16.04	50.30 ± 10.31 ^※※^	1.19 ± 0.08 ^※※^
Experimental group-7d	177.17 ± 12.59	60.32 ± 13.99 **	1.27 ± 0.10 **
Control group-14d	76.01 ± 4.68 ^※※^	82.44 ± 7.22 ^※※^	1.58 ± 0.26 ^※※^
Experimental group-14d	66.18 ± 6.54 ^&&^**	88.11 ± 9.17 **	1.75 ± 0.13 **

Note: At the same time, compared with the control group, ^&&^ *p* < 0.01, *p* < 0.05. In the same group, compared with the control group-3d, ^※※^ *p* < 0.01, *p* < 0.05. Compared with experimental group-3d, ** *p* < 0.01, *p* < 0.05.

**Table 5:** Area ratio occupied by collagen.

Group	Area Ratio
Control group-3d	20.56 ± 5.77
Experimental group-3d	21.05 ± 4.51
Control group-7d	24.52 ± 4.81
Experimental group-7d	25.57 ± 9.49
Control group-14d	30.64 ± 5.55 ^※※^
Experimental group-14d	40.97 ± 9.29 ^&^**

Note: At the same time, compared with the control group, *p* < 0.01, ^&^ *p* < 0.05. In the same group, compared with the control group-3d, ^※※^ *p* < 0.01, *p* < 0.05. Compared with experimental group-3d, ** *p* < 0.01, *p* < 0.05.

**Table 6:** Optical density value statistics table.

Group	Optical Density Value
Control group-3d	0.12 ± 0.03
Experimental group-3d	0.13 ± 0.04
Control group-7d	0.16 ± 0.03 ^※※^
Experimental group-7d	0.18 ± 0.03 **
Control group-14d	0.20 ± 0.02 ^※※^
Experimental group-14d	0.22 ± 0.03 **

Note: At the same time point, compared with the control group, *p* < 0.01, *p* < 0.05. In the same group, compared with the control group-3d, ^※※^ *p* < 0.01, *p* < 0.05. Compared with experimental group-3d, ** *p* < 0.01, *p* < 0.05.

## Data Availability

Data is contained within the article. The data presented in this study are available in tables and figures.
